# The Role of *Cryptococcus* in the Immune System of Pulmonary Cryptococcosis Patients

**DOI:** 10.1371/journal.pone.0144427

**Published:** 2015-12-04

**Authors:** Jinlin Wang, Yunxiang Zeng, Weizhan Luo, Xiaohong Xie, Shiyue Li

**Affiliations:** Department of Respiratory, The State Key Laboratory of Respiratory Disease, China Clinical Research Center for Respiratory Disease, Guangzhou Institute of Respiratory Disease, Guangzhou, Guangdong, China; The University of Texas at San Antonio, UNITED STATES

## Abstract

**Objectives:**

To investigate the role of *Cryptococcus* in the immune system of immunocompetent patients with pulmonary cryptococcosis (PC) by analysing the dynamic changes of patients’ immune status before and after antifungal therapy.

**Methods:**

The level of the serum interferon-γ (IFN-γ) and interleukin (IL)-2, -4, -10 and -12 was measured before and after 6-months of treatment. Peripheral blood samples were obtained from 30 immunocompetent PC patients and 30 age- and gender-matched healthy controls. Peripheral blood mononuclear cells (PBMCs) were isolated and incubated with recombinant human IL-12 (rhIL-12) for 48 h. Then the concentrations of IFN-γ and IL-4 in the supernatant were analysed.

**Results:**

Baseline serum IFN-γ level was significantly lower in the PC patients as compared with the control group (*P* < 0.001). The serum IL-2 and IFN-γ of PC patients were significantly increased after appropriate treatments (*P* < 0.05 and *P* < 0.001 when compared to their baseline levels). The productions of IFN-γ in the culture supernatant of PBMCs showed no significant difference between the control and PC patients both before and after antifungal treatments. RhIL-12 is a potent stimulus for IFN-γ production. Culture PBMCs collected from PC patients before treatments had a smaller increase of IFN-γ production in the present of rhIL-12 than the control (*P* < 0.01); PBMCs from PC patients completing 6-months of treatment showed a comparable increase of IFN-γ production by rhIL-12 stimulation to the control group.

**Conclusions:**

In apparently immunocompetent patients with PC, a normalization of serum IFN-γ was achieved after recovery from infection. This suggests that *Cryptococcus* infection per se can suppress the immune system and its elimination contributes to the reestablishment of an immune equilibrium.

## Introduction

*Cryptococcus neoformans (C*. *neoformans)*, an environmental yeast that causes pulmonary cryptococcosis (PC), is an opportunistic fungal pathogen that tends to infect immunocompromised hosts, especially those infected with human immunodeficiency virus (HIV)/Acquired Immune Deficiency Syndrome (AIDS) [1], the spread of *Cryptococcus* can result in meningitis which causes high morbidity and mortality [2]. In immunodeficient patients with PC, studies have revealed that deficiency in CD4^+^ T cells or the impaired anticryptococcal activity of monocytes will lead to reduced production of the protective cytokines and contribute to considerably high prevalence of cryptococcosis [3–5].

The host’s defence against *Cryptococcus* infection is mainly dependent on the T cell-mediated immunity (CMI) system; the host’s cytokines profile of immune system dramatically affects the outcome of PC [6–7]. Th1 and Th2 cytokines respond to *Cryptococcus* in a different manner. Th1-type cytokines, such as interferon-γ (IFN-γ) and interleukin (IL)-2 can enhance CMI and play a protective role in the host’s defence immunity against *Cryptococcus* [8–10]. By contrast, Th2-type cytokines, such as IL-4 and IL-10 having been reported as having a detrimental role for immunity. Kawakami K *et al*. [11] found that IL-4 suppressed the host defense mechanisms against infection with *C*. *neoformans* probably through the suppression of local production of IFN-γ. Murdock BJ *et al*. [12] revealed that early or late IL-10 blockade enhances Th1 and Th17 effector responses and promotes fungal clearance in mice with cryptococcal lung infection. Most of the *Cryptococcus* infection models showed that IL-12 plays a key role in the CMI system [13–15]. In the host, IL-12 is mostly produced by the macrophages and dendritic cells in response to *Cryptococcus*; IL-12 binds to the IL-12 receptor at the surface of T-lymphocytes and natural killer (NK) cells, thereby inducing Th0 cells to differentiate into Th1 cells [16]. Macrophages enhance cellular immunity and regulate the immune response chiefly *via* the IL-12/IFN-γ pathway [17].

However, the association between host’s immune system and *C*. *neoformans* infection is challenged by the recent increase in the number of PC cases in immunocompetent hosts. Up to 25% of the infectious cases in USA [18] and up to 53.9% cases in Shanghai Pulmonary Hospital [19] were diagnosed in patients without recognizable immunodeficiency. In our Institute, an increasing number of patients without comorbidity or immune dysfunction have been diagnosed with PC [20–21]. *Cryptococcus* possibly resides within the host permanently and the latent *Cryptococcus* may reactivate and cause the relapse of PC even after effective treatment [22–23]. Previous studies suggest that the mechanisms by which *Cryptococcus* was capable to infect healthy individuals may be inhibiting their protective immune response. But studies of cytokine response to *Cryptococcus* focused mainly on animal models [8–14] and immunocompromised patients with meningitis so far [5, 24–25]. Only a few studies have reported the interaction between *Cryptococcus* factors and the immunocompetent PC patients, as well as the modulation of adaptive immunity by this pathogen that leads to its subclinical persistence [22–23]. Our previous investigation on the changes in Th1/Th2 cytokines showed that an immune disturbance as manifested by reduced serum IFN-γ in immunocompetent PC patients [21]. But it is unclear whether the immune disorders are the cause or the consequence of the *Cryptococcus* infection. To further determine the role of *Cryptococcus* in PC patients’ immunity, dynamic changes of serum pro-inflammatory and anti-inflammatory cytokines, as well as the function of immune cells were investigated before and after antifungal treatment in current study.

## Materials and Methods

### Ethics Statement

The study design and protocol were approved by the Ethics Committee of the First Affiliated Hospital of Guangzhou Medical University. A written informed consent was obtained from each participant.

### Patients and diagnostic criteria

Thirty immunocompetent patients with PC were enrolled in the present study from March 2013 to June 2014 at the First Affiliated Hospital of Guangzhou Medical University. All patients met the diagnostic criterion for pulmonary cryptococcosis [19–20]: detection of *Cryptococcus* by cytological or histological identification in pulmonary resection specimens or in all kinds of lung tissues obtained by invasive puncture biopsy. Pathological diagnosis was independently evaluated by two experienced pathologists, when disputes occurred, a consensus was reached by discussion. According to clinical practice guidelines for management of cryptococcal disease by the Infectious Diseases Society of America (IDSA) [26], in nonimmunosuppressed PC patients, the stratages are different for mild-to-moderate or for severe patients. In our study, all the definitive patients were treated with fluconazole (400mg per day intravenously [IV]) for at least two weeks, administered by fluconazole (300mg per day orally) for at least six months. After 6 months appropriate therapy, patients were enrolled the study when chest CT scan revealed no abnormal findings. Thirty age- and gender- matched volunteers were also enrolled as controls. All control subjects received a physical check-up at our Medical Examination Centre within the same period and had a healthy history and no abnormalities in chest-ray or chest CT examination.

All included PC patients had negative determination of HIV antibodies, had a history of good health and absence of basic diseases (diabetes, chronic lung disease, ect). In addition, patients were excluded if they met the following criteria: 1) blood biochemical examination showed abnormal glucose, transaminase, serum creatinine, ect; 2) the peripheral absolute neutrophil count is <2.0 × 10^9^/L or the absolute lymphocyte count is <0.9 × 10^9^/L; 3) humoral immune parameters are below the lower limits of the normal range (IgG 6–16 g/L, IgA 0.7–5.0 g/L and IgM 0.6–2.0 g/L); 4) the patient is undergoing or had undergone immunosuppressant therapy; 5) the patient had undergone organ transplantation or exhibits graft-*versus*-host disease; 6) the patient received continuous administration of steroid hormones for more than three weeks; and 7) the patient exhibiting chronic diseases had been confined in the intensive care unit after surgery or trauma, was under long-term mechanical ventilation, exhibited an indwelling catheter in the body, received parenteral nutrition and was under long-term therapy with broad-spectrum antibiotics.

### Serum sample processing and cytokine immunoassays

When the definitive PC diagnosis was made, the immune status of the patient was assessed subsequently, considered as immunocompetent patient, serum samples were obtained before treating with fluconzole (which samples we labelled “before”). After the antifungal therapy for 6 months, chest CT scan showed no abnormal finding, serum samples were collected again (which samples we labelled “after”). Peripheral venous blood (10 mL) was extracted from the participants of both groups. The serum was obtained through low-speed centrifugation (2500 rpm) using a conventional desktop centrifuge (Eppendorf 5702; Germany), repacked and stored at −80°C. Enzyme-linked immunosorbent assay (ELISA) determined the serum concentration levels of IL-12, IFN-γ, IL-2, IL-4 and IL-10. The ELISA kits for human IFN-γ, IL-2, IL-4, IL-10 and human IL-12p70 were obtained from Dakewe Biotech Company, China.

### Supernatant cytokine immunoassays

Sterile heparinised peripheral venous blood (10 mL) was extracted from each participant in both groups. Following low-speed centrifugation at 2500 rpm (Eppendorf 5702; Germany), the blood plasma (upper layer) was removed, and lymphocyte isolation liquid (Ficoll-400, Dakewe Biotech Company, China) was added to the cells (lower layer). The PBMCs isolated from the whole blood were washed twice with RPMI 1640, and then resuspended in culture medium at a concentration of 1 × 10^6^ cells/mL. A 0.5 mL cell suspension was added into each well of a 24-well tissue culture plate. Subsequently, 0.5 mL RPMI 1640 was added into the wells of the control group, whereas 0.5 mL 20 ng/mL recombinant human (rh) IL-12 (ProSpec Biotech Company; USA) was added to the PC group. The plates were incubated in 5% CO_2_ incubator for 48 h at 37°C. Culture supernatants were harvested and assayed for IFN-γ and IL-4 levels by ELISA.

### Statistical analysis

All data were reported as means ± standard error. Statistical analysis was performed using SPSS. The two-sample two-tailed Student’s *t* test was used to compare numerical data among groups. Significance was set to *P* < 0.05 for all statistical analyses.

## Results

### Characteristics of the study population and controls

The 30 immunocompetent PC patients (PC group) enrolled in the study comprised 24 males and 6 females, with an average age of 37.70 ± 2.399 years old (in the range 20 years old to 65 years old). The characteristics of the controls did not differ from that of the PC group. All participants were in good health and without basic diseases or history of use of immunosuppressants.

In the PC group, the patients showed various symptoms of respiratory disease; up to 23.3% of the patients (*n* = 7) did not show any symptom, and their pulmonary diseases were incidentally detected during radiological examination. The symptoms of respiratory disease mainly included cough (60%,), productive sputum (16.7%), chest pain (10%), fever (6.7%) and hemoptysis (6.7%) (Table 1). No evident signs of respiratory diseases were observed through physical examination of the chest. In addition, the total white blood cell count, humoral immune parameters (including serum IgG, IgA and IgM) and serum CD4^+^ and CD8 ^+^ levels were normal in all PC patients.

**Table 1:** Demographic and clinical characteristics of the immunocompetent patients with PC.

Variable	All patients N = 30	Nodules N = 12	No-nodules N = 18	Surgeries N = 10	No-surgeries N = 20
Median age (years)	37.7	34.5	39.8	38.4	37.4
Male:female	24:6	9:3	15:3	7:3	17:3
No symptom (N)	7	5	2	4	3
Cough (N)	18	9	9	8	10
Productive sputum (N)	5	0	5	1	4
Chest pain (N)	3	0	3	1	2
Hemoptysis (N)	2	0	2	1	1
Fever (N)	2	0	2	0	2
VATS (N)	10	8	2	-	-
PBLB (N)	9	2	7	-	-
PCNB (N)	11	2	9	-	-

PC = pulmonary cryptococcosis; VATS = video-assisted thoracoscopic surgery; TBLB = transbronchial lung biopsy; PCNB = percutaneous cutting needle biopsy.

A chest CT scan was obtained from each patient before diagnosis. Based on their radiological features, chest lesions were classified into three patterns, as follows: 1) nodular lung mass type (12 patients, 40%), including 8 solitary nodular cases (26.7%) and 4 multi-nodular or mass cases (13.3%); 2) pneumonic type (8 patients, 26.7%); and 3) mixed types (nodular and pneumonic types) (10 patients, 33.3%). Cases of combined cavity (4 patients, 13.3%) and mediastinal lymphadenopathy (2 patients, 6.7%, non-surgical treatment group) were also observed. In our present study, we classified the patients into two subgroups, namely, nodules group (12 patients) and non-nodules group (18 patients) (Table 1).

Among 30 patients, 10 patients were diagnosed by VATS (surgical treatment group), where most of the lesions were removed at the time of diagnosis, and antifungal therapy was administered for 6 months [26]; the CT scan obtained after 6 months revealed the absence of any abnormality. The 20 other patients were diagnosed by TBLB (9 patients) and percutaneous cutting needle biopsy (11 patients) (Table 1). Antifungal therapy was implemented after diagnosis, the CT scan conducted after 6 months revealed no abnormal findings.

### Comparison of the baseline cytokine response during PC in immunocompetent host and controls

**Fig 1:**
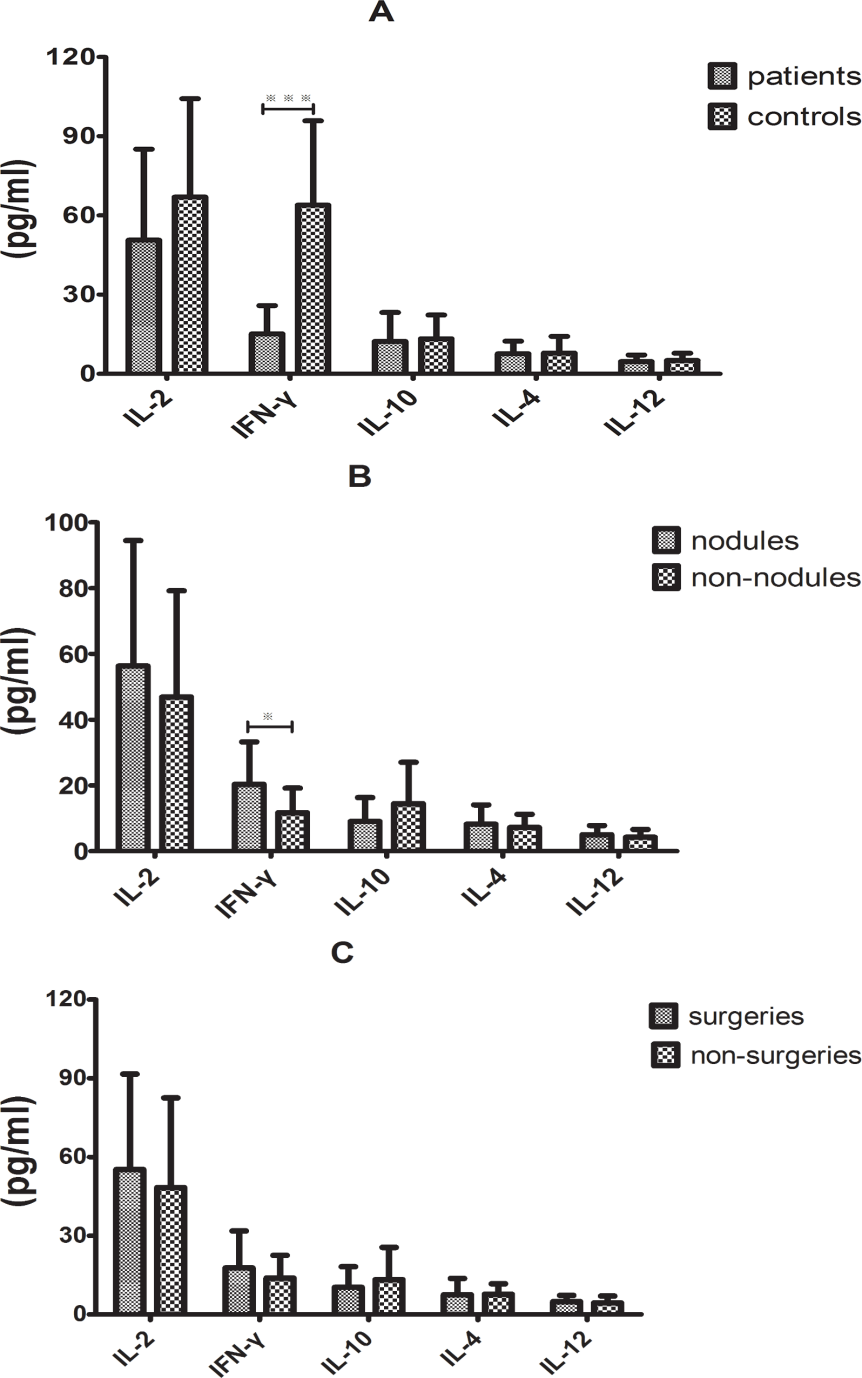
Baseline cytokine response in immunocompetent PC host compared with the controls. (A)Serum IFN-γ level was significantly reduced in the PC patients (***P < 0.001). No significant differences in IL-2, IL-10, IL-4 and IL-12 levels were observed between the two groups. (B) Serum IFN-γ levels was higher in the nodules subgroup than in the non-nodules subgroup (*P < 0.05); similar levels of the other cytokines was observed in the two subgroups. (C) Serum IFN-γ levels was higher in the surgical treatment subgroup than in the non-surgical subgroup; the amounts of the other cytokines were similar in the two subgroups.

The cytokine levels in the baseline serum of the PC group and the control group were initially compared using univariate analyses. The average levels of serum IL-2, IFN-γ, IL-10, IL-4 and IL-12 in the PC group compared with the control group were 50.632 ± 6.29 pg/mL *v*.*s*. 66.973 ± 6.80 pg/mL, 15.140 ± 1.95 pg/mL *v*.*s*. 63.880 ± 5.84 pg/mL, 12.276 ± 2.00 pg/mL *v*.*s*. 13.297 ± 1.640 pg/mL, 7.614 ± 0.87 pg/mL *v*.*s*. 7.851 ± 1.15 pg/mL and 4.621 ± 0.46 pg/mL *v*.*s*. 5.084 ± 0.50 pg/mL, respectively. The serum IFN-γ concentrations were significantly lower in the PC group than in the control group (*P* < 0.001), whereas the serum levels of the other cytokines were similar in the two groups (Fig 1A).

Subgroup analysis revealed that the serum IFN-γ level was higher in the nodules subgroup than in the non-nodules subgroup (*P* < 0.05), whereas the serum levels of the other cytokines did not significantly differ between the two subgroups (Fig 1B). But between the surgical treatment subgroup and the non-surgical treatment subgroup, there were no significant differences no matter the serum IFN-γ level or other serum cytokines (Fig 1C).

### Supernatant IFN-γ and IL-4 productions after stimulating PBMCs *in vitro* with rhIL-12 in baseline

**Fig 2:**
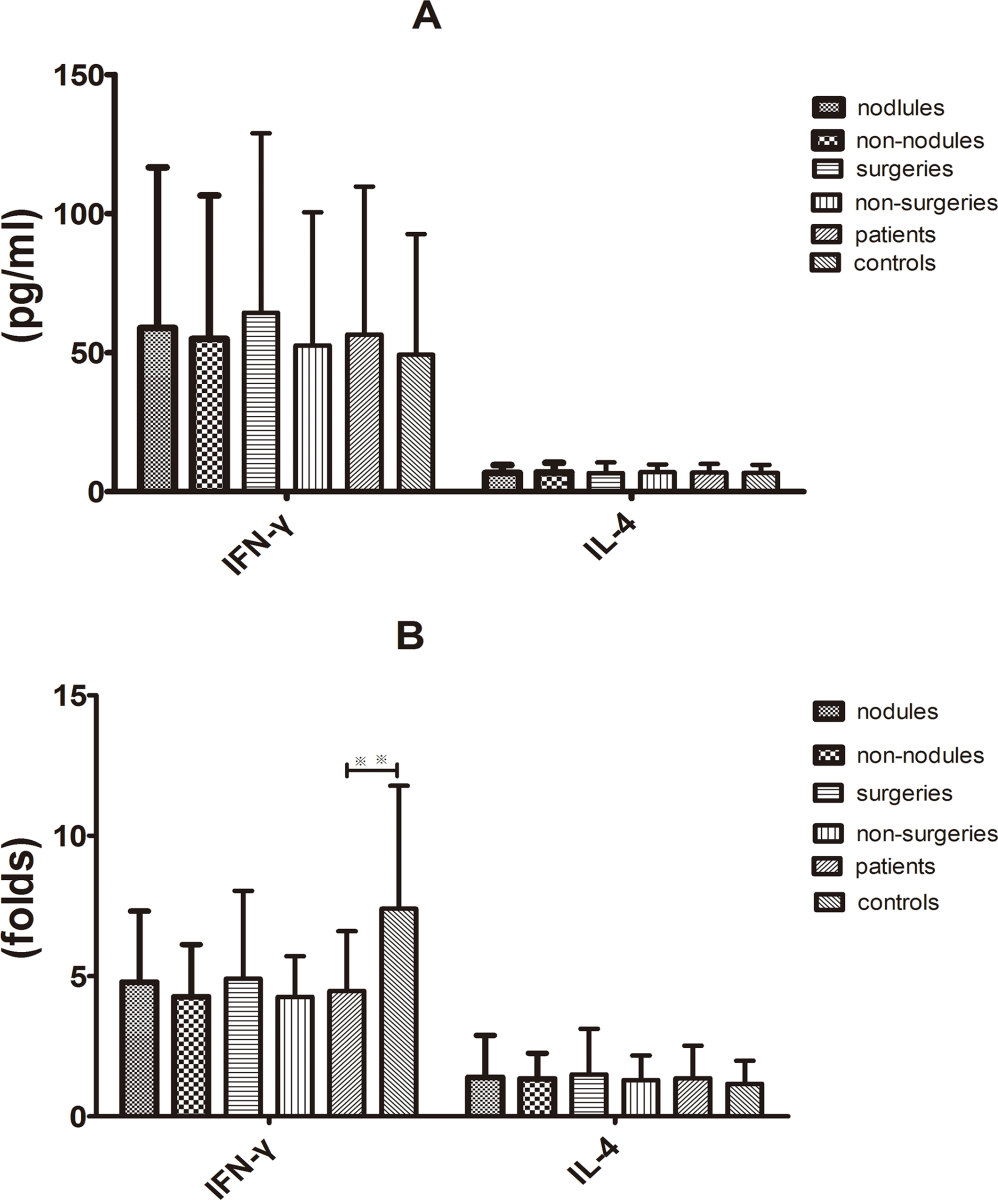
Baseline supernatant IFN-γ and IL-4 levels in rhIL12-stimulated PBMCs *in vitro*. (A) Concentrations of IFN-γ and IL-4 in the supernatant of PBMCs without rhIL12 stimulation was similar between the patients and controls, between the nodules and non-nodules subgroups and between surgical and non-surgical treatment subgroups. (B) rhIL-12 stimulated the release of IFN-γ; increase in IFN-γ level was lower in the patients (4.47-fold) than in the controls (7.40-fold) (**P < 0.01); rhIL-12 did not stimulate IL-4 secretion in the supernatant of PBMCs in both groups. No differences in IFN-γ or IL-4 levels were observed between the nodules and non-nodules subgroups and between surgical and non-surgical treatment subgroups.

IL-12-activated T cells of the blood directly affect *C*. *neoformans* [17]. To establish the anticryptococcal effect and determine the effector mechanisms that were induced by IL-12, PBMCs were stimulated with rhIL-12. Without rhIL-12 stimulation, the average levels of the supernatant IFN-γ and IL-4 in the PC and control groups were 56.519 ± 9.71 pg/mL *v*.*s*. 49.239 ± 7.92 pg/mL and 6.882 ± 0.57 pg/mL *v*.*s*. 6.755 ± 0.53 pg/mL, respectively. The two groups showed relatively similar average concentrations of the supernatant IFN-γ and IL-4 (Fig 2A). Following PBMCs stimulation with rhIL-12 *in vitro*, the IFN-γ levels increased in the PC and control groups (4.47 ± 0.39 folds *v*.*s*. 7.40 ± 0.80 folds) and the increment of change in PBMCs was much lower in the PC than in the control group (*P* < 0.01). By contrast, no significant difference between the two groups was observed in terms of the increment of change in the IL-4 levels (1.36 ± 0.21-fold *v*.*s*. 1.16 ± 0.15-fold) (Fig 2B). In addition, no significant difference in the baseline supernatant IFN-γ levels, IL-4 levels and increment of change in IL-4 levels was observed between the nodules and the non-nodules subgroups and between the surgical treatment and non-surgical treatment subgroups (Fig 2A and 2B).

### The influence of effective therapy on the serum cytokine response in immunocompetent PC patients

**Fig 3:**
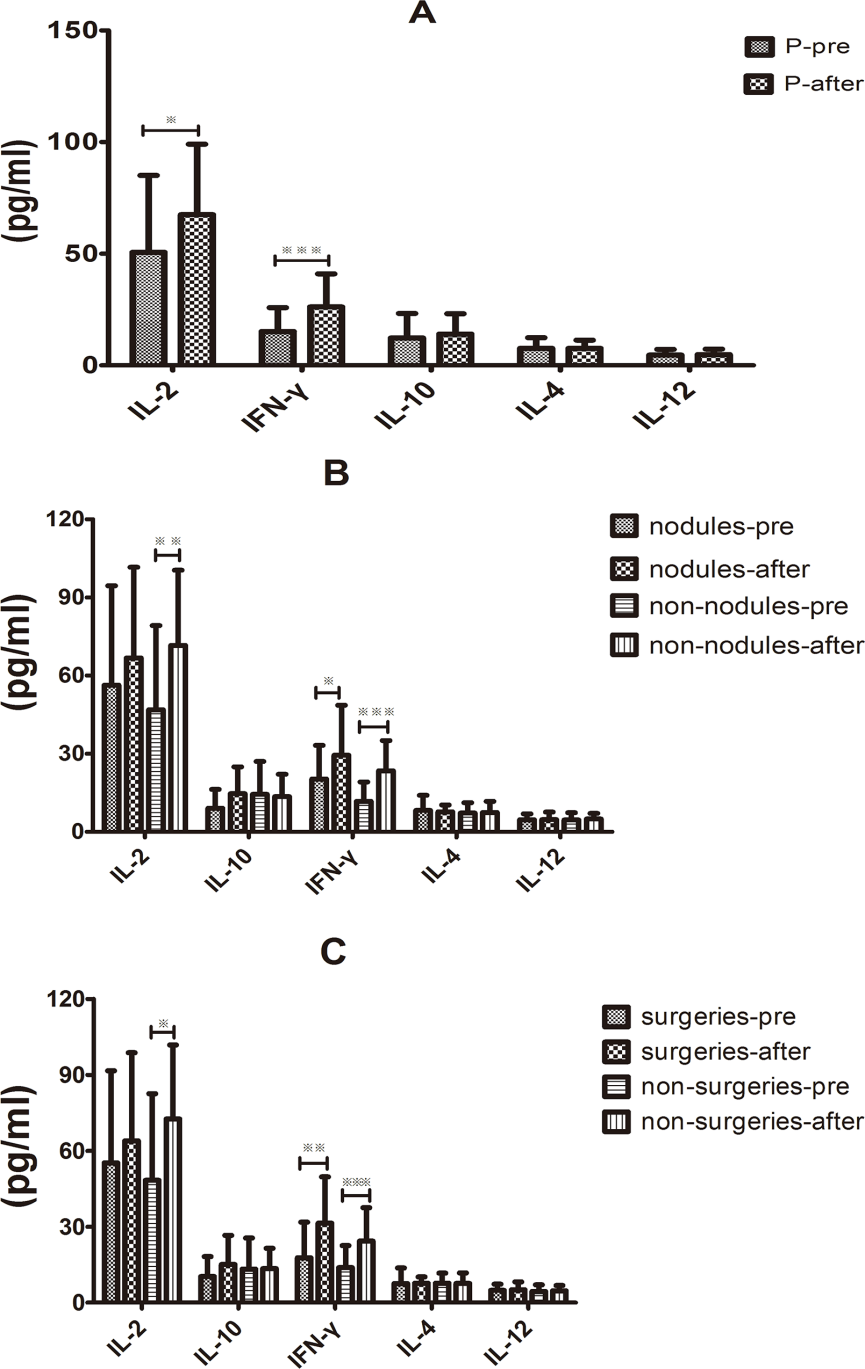
Influence of the effective therapy on the serum cytokine response in immunocompetent PC patients. (A) Average levels of the serum IL-2 and IFN-γ were significantly higher following effective therapy than at baseline (*P < 0.05 and **P < 0.001), whereas IL-4, IL-10 and IL-12 levels were similar to baseline. (B) Serum IL-2 and IFN-γ levels were significantly increased in the non-nodules group compared with the baseline (**P < 0.01 and ***P < 0.001); in the nodule group, only the IFN-γ levels were much higher compared with the baseline (*P < 0.05); the effective therapy did not affect the secretions of IL-10, IL-4 and IL-12. (C) Serum IL-2 and IFN-γ levels were significantly increased in the non-nodules group compared with the baseline (**P < 0.01 and ***P < 0.001); in the nodules group, only IFN-γ levels were much higher compared with the baseline (*P < 0.05); the effective therapy did not affect the secretion of IL-10, IL-4 and IL-12.

After 6 months of effective therapy, no abnormal findings were found based on the chest CT scan results of the PC patients. The dynamics of the serum cytokine response in PC patients were examined to determine whether the serum cytokine responses in the immunocompetent PC patients differed from controls after recovery from infection. The average serum levels of IL-2 and IFN-γ were 67.507 ± 5.758 pg/mL *v*.*s*. 50.632 ± 6.29 pg/mL and 26.147 ± 2.711 pg/mL *v*.*s*. 15.140 ± 1.95 pg/mL, respectively, which were significantly higher after the effective therapy compared with the baseline cytokine levels (*P* < 0.05 and *P* < 0.001); whereas, the serum levels of IL-4, IL-10 and IL-12 were similar to that of the baseline (Fig 3A). Interestingly, further subgroup analysis on serum cytokines revealed that IL-2 and IFN-γ were significantly increased in non-nodules group compared with baseline (*P* < 0.01 and *P* < 0.001); in the nodules group, only the IFN-γ levels were much higher than baseline (*P* < 0.05). Recovery from infection did not affect the other cytokines, including IL-10, IL-4 and IL-12 (Fig 3B). Similar results were seen in both surgical treatment and non-surgical treatment groups (Fig 3C).

### Supernatant levels of IFN-γ and IL-4 in rhIL-12-stimulated PBMCs *in vitro* after effective therapy

**Fig 4:**
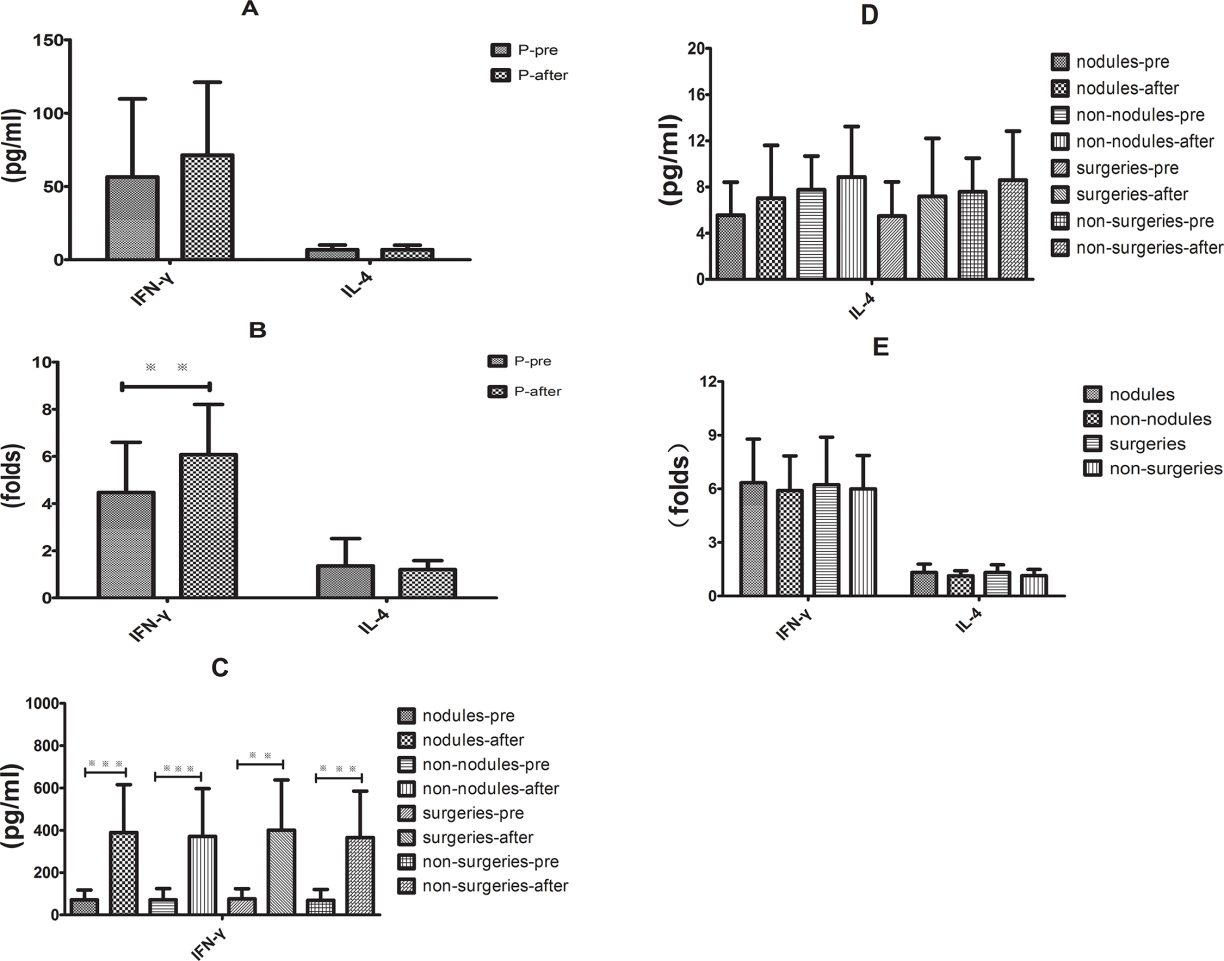
Supernatant IFN-γ and IL-4 levels in rhIL-12-stimulated PBMCs *in vitro* following the effective therapy. (A) Supernatant IFN-γ and IL-4 levels after the effective therapy were not significantly different from the baseline. (B) rhIL-12 stimulation significantly increased the median levels of supernatant IFN-γ compared with the baseline (**P < 0.01). (C) Supernatant IFN-γ level was much higher in different subgroups compared with the baseline (**P < 0.01 and ***P < 0.01). (D.E) No significant difference in the supernatant IL-4 level and increment of changes in IL-4 was observed following the effective therapy compared with baseline.

PBMCs were isolated from the PC patients and controls, and thes cells were stimulated ex vivo with rhIL-12. The results revealed that the supernatant IFN-γ level was similar to that of the baseline, with rhIL-12 stimulation, the median levels of supernatant IFN-γ was greater by 6.08 ± 0.39-fold compared with the baseline (*P* < 0.01). But there was no significant difference in the supernatant IL-4 levels and increment of changes in IL 4 between the baseline and after rhIL-12 stimulation (Fig 4A and 4B).

To analyse the different responses to treatment, we divided the groups into different subgroups. Compared with baseline, we found that supernatant IFN-γ was much higher than baseline in the different subgroups (*P* < 0.01) (Fig 4C) but no significant differences were found for supernatant IL-4 (Fig 4D). In addition, the increment of change in the supernatant IFN-γ and IL-4 levels were similar between the nodules and the non-nodules groups and between the surgical treatment and non-surgical treatment groups (Fig 4E).

## Discussion

Previous studies on cytokine response in cryptococcosis patients focused mostly on immunocompromised patients or those with meningitis [3–5, 25, 27]. They suggested that immune deficiency, such as those caused by CD4^+^ T cell deficiency or impaired anticryptococcal activity of monocytes would reduce the protective cytokine levels, which possibly contribute to the low clearance of *C*. *neoformans* in PC patients [3, 5]. Zheng CF *et al*. [3] further revealed that CD4^+^ and CD8^+^ lymphocytes performed the anticryptococcal function by granulysin that was expressed upon STAT5 and PI3K activation in the presence of IL-2/IL-15 and IL-15, respectively. In HIV patients, these two pathways are defective, thereby resulting in inefficient *C*. *neoformans* elimination.

However, the interaction between *C*. *neoformans* and immune system in the immuocompetent PC patients was poorly known. The patients enrolled in the present study were initially in good health condition, did not suffer from basic diseases and had no history of immunosuppressant use. Laboratory examinations showed that their white blood cells, neutrophils, lymphocytes, serum immune globulins and serum CD4^+^ and CD8^+^ levels were all within the normal range. At present, there are currently no unified criteria to evaluate the immune function of PC patients, the practical assessment is always a comprehensive consideration of the presence or absence of basic diseases, a history of using immunosuppressive drugs, the total count and classification of white blood cells, humoral immune parameters, serum CD4^+^ and CD8^+^ levels, etc [19–21, 28–29]. Therefore, the enrolled patients were considered as immunocompetent.

We compared the anti-inflammatory cytokines profile of the 30 immunocompetent PC patients with those of the controls and found that the baseline serum IFN-γ level was significantly lower in the immunocompetent PC patients than in the controls. We also found that production of IFN-γ and IL-4 in the supernatant of culture PBMCs were similar in both PC and control groups (*P* > 0.05). However, rhIL-12 stimulation lead to a much lower increase of IFN-γ production in the supernatant of the culture PBMCs from PC patients when compared to those of control group (4.7 folds *v*.*s*. 7.4 folds). It indicated that a certain mechanism which may be responsible for the decreased secretion of IFN-γ by immune cells. This observation is consistent with our previous finding [21], but it is still unclear whether these “immunocompetent” individuals actually have hidden immunocompromising conditions and that the abnormities of cytokines profiles predate the PC infection.

To further determine if PC infection leads to this immune disturbance, we analysed the same serum cytokines in PC patients after 6 months of antifungal treatment. Interestingly, the serum IL-2 and IFN-γ levels in the PC group were significantly increased as compared to its baseline. Moreover, the increase of IFN-γ production due to rhIL-12 stimulation in the supernatant of the PBMCs culture medium was also significantly larger than those in the baseline (6.08 folds *v*.*s*. 4.7 folds). The improved reactivity and sensitivity to the IL-12 stimulation of immune cells is possibly one of underlying mechanisms for the increased serum level of IFN-γ in the PC patients completing antifungal treatment. Considering that there is little evidence about a positive role of antifungal medicine on host’s immunity, the restoration of a protective immunity after treatment may be due to the clearance or recovery from PC infection. This also suggests that the immune disorders manifested by abnormal anti-inflammation cytokines in PC patients at the time of diagnosis result from the PC infection.

Cheng PY *et al*. [30] found in a mice experiment that *C*. *gattii* strains appeared to reduce the cytokine levels in immunocompetent mice. Brouwer AE *et al*. [31] observed that HIV-negative patients with cryptococcal meningitis similarly exhibited reduced levels of the cytokines IFN-γ, TNF-α and IL-6 compared with patients with HIV-related *C*. *neoformans* infections. These observations suggested that the patients exhibited a maladaptive immune response to cryptococcal exposure, which had allowed progression to clinical cryptococcal disease. The impact of *C*. *neoformans* infection on the development of the adaptive T helper cell immune response has not been fully addressed. Cheng PY *et al*. [30] suggested that *C*. *gattii* infection could dampen pulmonary neutrophil recruitment and inflammatory cytokine production in immunocompetent hosts. In addition, Angkasekwinai P *et al*. [32] recently revealed that *C*. *gattii* infection dampens the DC-mediated effective Th1/Th17 immune responses and downregulates pulmonary chemokine expression, thereby resulting in the loss of protective immunity in immunocompetent hosts. Huston SM *et al*. [33] reported that TNF-α did not increase in the presence of *C*. *gattii*; however, the addition of recombinant TNF-α or stimulation that led to TNF-α production restored DC maturation and T cell responses. Thus, *C*. *neoformans* exhibits the ability to develop adaptive immunity, thereby infecting immunocompetent individuals. Current study is the first to investigate the dynamics of serum cytokine production in immunocompetent PC patients and show an important role of cryptococcosis in the immune system of immunocompetent PC patients. Cryptococcosis can occur not only in immunocompromised patients but also down-regulate the immunity of a healthy subject and contribute to its infection progression in the immunocompetent patient. Further studies are needed to elucidate the mechanisms of downregulation of cytokine production during *C*. *neoformans* infection.

A substantial effect of *C*. *neoformans* on the host’s immune systems was further proved by estimating the cytokine profile in different subgroups of PC patients (nodular *v*.*s*. non-nodular; surgery *v*.*s*. non-surgery). Immunocompetent patients with PC infection are usually minimally symptomatic or even asymptomatic. Infection in some patients is only incidentally detected during a regular physical examination by radiologic technique, and the nodular type is more common than the others, especially in immunocompetent patients. On the contrary, immunocompromised hosts tend to exhibit the pneumonic and mixed types, although the pneumonic type is more frequent [19, 28, 34]. Thus non-nodular pathological type may indicate a higher burden of PC infection than the nodular type [35]. Different types of lung lesions are possibly associated with the status of immune response even in immunocompetent patients. To the best of our knowledge, the relationship between the immune status of immunocompetent PC patients and lesion types has not yet been reported. Thus, we compared the antiflammatory cytokine profile in nodules subgroup and non-nodules subgroup of PC patients. In addition, some of the immunocompetent PC patients were diagnosed by occasional surgery who presented as solitary nodule or mass preoperatively diagnosed as lung cancer. After the surgery, antifungal drugs and duration were currently no consensus, some scholars consider that the fungi-containing lesions, which will gradually develop into granulomas, manifested radiologically as solitary or multiple nodular lesions in immunocompetent host, would be removed almost and eliminate the *C*. *neoformans* burden in surgical patients, so they even did not need to accept antifugal drugs [35]. In our study, most of the lesions were removed without mediastinal, hilar and interlobar lymphadenectomies when the frozen sections obtained from the lesion intraoperatively showed benign result, six months antifugal drugs were followed-up in our surgical patients according to practice guidelines [26]. We also compared the immune status to preliminary understand the cytokine profile in in surgical and non-surgical treatment subgroups as well.

Subgroups analysis revealed that serum levels of IFN-γ were higher in the nodules subgroup which than in the non-nodules subgroup which has a higher burden of PC infection (*P* < 0.05). The serum levels of IFN-γ and IL-2 were obviously increased in the non-nodules but only slightly increased in the nodules groups after antifungal treatment, indicating a close relationship between PC elimination and immunity restoration.

Intersting, surgical *v*.*s*. non-surgical treatment subgroups, similar difference was observed as in nodular *v*.*s*. non-nodular subgroups. The explaination for the consistent results in different subgroups may be that the surgeries were mostly performed in nodular lung mass cases. The interesting results may be one of the explaination for that nodular lung mass was more common in immunocompetent patients compared with immunocompromised hosts [19, 28, 34].

In conclusion, the serum IFN-γ levels were reduced in immunocompetent PC patients, especially those with nodular pathological lesion. After antifungal treatment, the serum IFN-γ level was significantly increased due to improved reactivity and sensitivity of immune cells to IL-12 stimulation, which apparently restored the protective inflammatory response of the host’s immune systems. Taken together, *Cryptococcus* can not only infected the immunocompromised patients but also affect the immunity of a healthy subject and contribute to its infection progression in the immunocompetent patient.

## Supporting Information

S1 Dataset Detailed figures of serum or supernatant cytokines of PC patients. (XLS)
